# Furniture for Nurses' Rooms

**Published:** 1893-07-01

**Authors:** 


### PRACTICAL DEPARTMENTS.

### FURNITURE FOR NURSES' ROOMS.

In considering the best kind of furniture for nurses' rooms the shape and size of the room should be taken into account first of all. It is, of course, essential that furniture which is intended strictly for use and not for ornament should be simple in make, with as few dust traps as can be managed, and as nurses' quarters in all but a few favoured exceptions are necessarily limited in space, all fittings should be as compact as possible, while at the same time as commodious as space will allow. In most nursing homes the cubicles or small rooms allotted to the members of the nursing staff are only capable of holding a bed, chest of drawers or wardrobe, and washstand, in addition to a linen basket and a chair. Yet much can be done even within these narrow limits to make a nurse's bedroom cosy and comfortable, provided its contents are well adapted to the requirements of the occupier, and no little rooms could well look more comfortable or more home-like than some we have seen in the nursing homes of our large hospitals.

Before proceeding to discuss the arrangements in detail, we should like to suggest that an idea for "combination furniture," as described in a small handbook on "Healthy Furniture and Decoration," written by Robert W. Edis, F.S.A., and published at the time of the Healtheries Exhibition, might with advantage be taken into consideration where new nursing homes are contemplated. The notion is that one side of a bedroom shall be fitted up with wardrobe, drawers, bookshelves, boot cupboards, "all designed as part of the internal furnishing of the room, carried up to the ceiling line, with top cupboards for store places for clothing which has to be put away for a season. In this way boots and bottles, and various odds and ends which accumulate in every room, encumbering and littering the floor space . . and forming at all times unhealthy and objectionable items in a room, and rendering it at the same time difficult to keep clean and tidy, might easily be put away, to the manifest comfort of the occupier and the greater healthiness of the room." Nurses' rooms, forming as they do parts of public institutions, have necessarily to be kept absolutely free from the "muddles" which often distinguish the sleeping rooms of private individuals, but with the limited accommodation allotted to them nurses may often well be puzzled to stow away their belongings in a satisfactory manner. If we think of it, much valuable space is lost at the top of the room, with ordinary movable furniture, which would be utilized with much advantage were this arrangement of permanent fittings carried out.

Deep drawers might form the lowest portion of these fittings, while the centre part is divided into hanging cupboard with glass panelled door, thus avoiding the necessity of a separate dressing glass, and "in the other part arranged with . . boot cupboards, enclosed trays and shelves for linen . . and the whole might be carried out in plain deal, stained and varnished, or painted and varnished, to match the other woodwork."

Rooms fitted up in such a manner would be far less trouble to clean, and thus considerable saving of labour would be the result, always a most desirable end to keep in view in the furnishing of any part of an institution where rigid economy in such details is essential, besides rendering the room itself far healthier from a sanitary point of view, inasmuch as all those dust-traps which exist where the furniture is movable, i.e, narrow spaces between it and the skirting board, where dust and dirt collect in a manner that is quite astonishing, would be entirely done away with.

In furnishing very small rooms it is a pity that corner cupboards or washstands are not more frequently in request. These also afford much saving of space and are in every way most convenient. It is a very apparent fact that unused corners are wasted accommodation, and where it is not considered desirable to fit up the whole of a side of a room, why should not a couple of corners be used, one for the washstand and the other for a hanging closet, the latter carried up to the ceiling, the top space being used as a storage cupboard? In this way there will be more of both air and floor space, advantages which are very important in providing healthy sleeping rooms for nurses, who naturally need as much of both as is possible.

In a future article we shall hope to be enabled to give a sketch of a nurse's bed-room, arranged in the way we have described, a way which should, we think, commend itself to the authorities of nursing institutions.

